# Supplementary material for: Nurses’ Role in Obesity Management in Adults in Primary Healthcare Settings Worldwide: A Scoping Review
Source: Healthcare (Basel). 2024 Aug 26;12(17):1700. doi: 10.3390/healthcare12171700 (PMC11395003; doi:10.3390/healthcare12171700)
Supplement: Supplementary file 1 [file healthcare-12-01700-s001.zip › healthcare-3171077-supplementary.pdf]

Table S1. Extracted data items related to nurses' role in obesity management.

Date	Concept Block	Selected Search	Results
	**CINAHL via EBSCOhost**		
**April 30, 2024**	S3: Obesity (S1 OR S2)<br>CINAHL HEADINGS S1: (MH "obesity") OR (MH "Obesity, Morbid") Keywords S2: TI (obes* or "high BMI" or "high body mass index") OR AB (obes* or "high BMI" or "high body mass index")	S14: S6 AND S12 AND S13<br><br>Nursing Role AND nursing AND (obesity OR weight management)<br><br>Limiters Published Date: **January 01, 2018–April 30, 2024**	**N = 331**
	S6: Nursing Role (S4 OR S5)<br>CINAHL HEADINGS S4: (MH "role") OR (MH "Nursing Role") OR (MH "Nursing Interventions")<br>Keywords S5: TI (role* or "nurs* role*" or "nurs* function*" or "nurs* strateg*" or "nurs* intervention*") OR AB (role* or "nurs* role*" or "nurs* function*" or "nurs* strateg*" or "nurs* intervention*")		
	S9: Weight management (S7 OR S8)<br>CINAHL HEADINGS S7: (MH "weight control") OR (MH "Weight Reduction Programs") OR (MH "weight management (Iowa NIC)")<br>Keywords S8: TI ("weight control" or "weight reduction" or "weight management") OR AB ("weight control" or "weight reduction" or "weight management")		
	S12: Nursing (S10 OR S11)<br>CINAHL HEADINGS S10: (MH "nurses")<br>Keywords S11: TI (nurse* or nursi* or "nurse-directed" or "nurse-led" or "nurse-managed") OR AB (nurse* or nursi* or "nurse-directed" or "nurse-led" or "nurse-managed")		
	S13: S3 OR S9<br>obesity OR weight management		
	**MEDLINE via EBSCOhost**		
**April 30, 2024**	S3: Obesity (S1 OR S2)<br>MeSH S1: (MH "obesity") OR (MH "Obesity, Morbid") OR (MH "obesity management")<br>Keyword S2: TI (obes* or "high BMI" or "high body mass index") OR AB (obes* or "high BMI" or "high body mass index")	S14: S6 AND S12 AND S13<br><br>Nursing Role AND obesity OR weight management AND nursing	**N = 362**
	S6: Nursing role (S4 OR S5)<br>MeSH S4: (MH "role") OR (MH "Professional Role") Or (MH "nurse's role")<br>Keywords S5: TI (role* or "nurs* role*" or "nurs* function*" or "nurs* strateg*" or "nurs* intervention*") OR AB (role* or "nurs* role*" or "nurs* function*" or "nurs* intervention*")		
	S9: Weight management: (S7 OR S8)		

	MeSH S7: (MH "Body Weight Maintenance") OR (MH "Body Weight Changes") OR (MH "weight loss+") OR (MH "Weight Reduction Programs+")<br>Keywords S8: TI ("weight control" or "weight reduction" or "weight management") OR AB ("weight control" or "weight reduction" or "weight management")	Limiters<br>Published Date:<br>**January 01, 2018–April 30, 2024**	
	S12: Nursing (S10 OR S11)<br>MeSH S10: (MH "nurses") OR (MH "Practice Patterns, Nurses'") OR (MH "Delivery of Health Care")<br>Keywords S11: TI (nurse* or nursi* or "nurse-directed" or "nurse-led" or "nurse-managed") OR AB (nurse* or nursi* or "nurse-directed" or "nurse-led" or "nurse-managed")		
	S13: S3 OR S9<br>obesity OR weight management		
	**PsycInfo (via EBSCO)**		
**April 30, 2024**	S3: Obesity (S1 OR S2)<br>Descriptors S1: DE "obesity" OR DE "body weight" OR DE "overweight" OR DE "weight control" OR DE "weight loss" OR DE "body mass index" OR DE "obesity (Attitudes Toward)"<br>Keyword S2: TI (obes* or "high BMI" or "high body mass index") OR AB (obes* or "high BMI" or "high body mass index")	S11: S9 AND S10<br><br>nursing AND obesity OR weight management<br><br>Narrow by subject age: thirties (30-39 yrs), young adulthood (18-29 yrs), middle age (40-64 yrs), adulthood (18 yrs and older)<br><br>Limiters<br>**January 01, 2018–April 30, 2024**	**N = 449**
	S6: Weight management (S4 OR S5)<br>Descriptors S4: DE "weight loss" OR DE "weight control"<br>Keywords S5: TI ("weight control" or "weight reduction" or "weight management" or "overweight") OR AB ("weight control" or "weight reduction" or "weight management" or "overweight")		
	S9: Nursing (S7 OR S8)<br>Descriptors S7: (DE "nurses" OR DE "public health service nurses") OR (DE "nursing")<br>Keywords S8: TI (nurs* or "nurs* intervention*" or "nurs* strateg*" or "nurs* role" or "nurs* guided" or "nurse-directed" or "nurse-led" or "nurse-managed") OR AB (nurs* or "nurs* intervention*" or "nurs* strateg*" or "nurs* role" or "nurs* guided" or "nurse-directed" or "nurse-led" or "nurse-managed")		
	S10: S3 OR S6<br>obesity OR weight management		

Table S2. Key descriptive characteristics of the included documents.

First Author/ Year/ Citation	Aim	Key Findings: Nurses' Roles in Obesity Management	
Barrea (2021) [1]	"To provide practical guidelines for nurses working in Obesity Clinic for effective management of obesity and its related diseases."	**Education**<br>-Health and lifestyle education.<br>**Interventions**<br>-Assessment of social determinants of health, medical history, physical and psychological exam, and lifestyle habits.<br>-Therapeutic relationship development.<br>-Behavioural therapy.<br>-Goal setting.<br>-Caring for related diseases.	**Monitoring**<br>- Medication side effects.<br>-Comorbidities.
Braga (2020) [2]	"To understand the actions of nurses toward obesity in primary health care units."	**Patient-centred care**<br>- Motivate / empower / support.<br>- Assess clinical setting (lacking basic material, scales).<br>- Build therapeutic relationships.<br>- Team-based care (multidisciplinary).<br>**Patient assessments**<br>- Anthropometric measurement (weight/ heigh/BMI, WC).<br>- Lifestyle factors (nutrition).<br>- Physical (comorbidities, diagnoses, BP, health risks).<br>**Therapeutic interventions**<br>- Treatment options (lifestyle/ behavioural change, metabolic surgery).<br>- Approach (SDOH-focused).<br>- Measures of success (body size, lifestyle/ behavioural change).	**Case management**<br>- Coordinate.<br>- Program development.<br>- Schedule/ follow-up.<br>- Monitor progress.<br>- Refer.<br>**Patient education**<br>- Medication.<br>- Physical activity.<br>- Nutrition.<br>**Professional practice**<br>- Reflexivity (gaps in knowledge).<br>- Ongoing learning (available guidelines, obesity education).

Brewah (2018) [3]	The aim of this paper was to address the following question: " Can community nurses take on obesity?"	**Advocacy** (for healthier community environment)<br>- Healthier access to food, drinks, and physical activity in the community.<br>**Initiation of first conversation about obesity management**<br>- Asking permission, using open-ended questions, supporting individuals' change readiness and agenda setting.	**Education**<br>- Nutritional and alcohol intake, local fitness classes, and online resources.<br>**Interventions**<br>- Evidence-based care.<br>- Individualized approach to national guidelines.
Campbell-Scherer (2019)* [4]	"The objective of the 5AsT trial was to assess whether a co-created educational intervention would increase the quantity of obesity visits conducted by chronic disease nurses in a family practice."	**Initiation of first conversation about obesity management**<br>-Providing evidence based-care.<br>**Promotion of the 5As framework**<br>-Provider knowledge and confidence.<br>-Views of obesity management.<br>-Role identity.<br>-Interprofessional relationships.<br>-Relationships with patients.<br>See Ogunleye et al. (2015) below for topics covered in each session in the 5As education sessions.	
Fernández- Ruiz (2018) [5]	"The aim of the study was to test if using an interdisciplinary programme based on healthy eating, physical exercise, and cognitive behavioural therapy coordinated by nurses could improve anthropometric and cardiovascular measures related to obesity in the short, medium, and long term."	**Multidisciplinary team coordination**<br>-Goal setting/establishing intervention targets and indicators.<br>-Serving as link between patients and the team.<br>**Education**<br>-Participating in education program development and delivery for staff and patients.<br>**Advocacy and navigation (for the patient)**<br>-Attending multi-disciplinary activities with the patient.<br>-Advocating for patients and/or patient participation in team meetings.	

		- Closely supporting patients throughout the whole program.<br>-Ensuring individualized planning, coordination, follow-up, and contact.	
Fernández-Ruiz (2018) [6]	"The main objective of this study is to determine the effectiveness of the I2 AO2 (Interdisciplinary Intervention Against Overweight and Obesity) program on HRQoL and psychological comorbidity in the medium (12 month) and long (24 month) term… The second objective of this study is the implementation of this program under the coordination of nursing."	**Multidisciplinary team coordination**<br>-Leadership: "empower the patient's autonomous change and establish a link between the different professionals, the community and the community patient."<br><br>See the previous article by the same author for perspectives of both papers on nurses' roles.	
Govindasamy (2023) [7]	"The aim of this paper was to test whether it is feasible and acceptable to motivate patients from a disadvantaged background to initiate and maintain dietary regulation, by task shifting to PNs [practice nurses'] within a general practice setting. "	**Interventions**<br>- Assisting patients to set dietary goals.<br>-Assisting patients with completing and interpreting food diaries.<br>- Assisting patients with identifying obstacles to change and practical ways to overcome them.<br>-Assisting patients with developing comprehensive action plans—with regular review.<br>-Following up with patients every 2 weeks for 8 weeks.<br>-Providing emotional support and motivation.	
Hinks (2022) [8]	"The aim of this research was to understand community nurses' views on weight management within their practice and the potential implementation of a WMP	**Education for patients**<br>-Nutrition, chronic health management (e.g., diabetes, wound care), mental health well-being, and additional topics, such as smoking cessation.<br>**Education for providers**<br>-Using the weight management pathway.	

	[Weight Management Pathway]."	
Kelley (2018) [9]	Faith community nurses support lifestyle modification and offer spiritual support to those with obesity.	**Promoting the 5As framework**<br>-Using the 5As tool for behaviour change (assess, advise, agree, assist, arrange).<br>- Assessing weight, height, and BMI and inform the individual of the diagnosis.<br>- Advising the individual by counselling to decrease calories and increase regular exercise.<br>Once an individual agrees there is a weight concern and agrees to care, then the following is completed:<br>- Assisting with developing a care plan that focuses on individual strengths, past success, and identified areas of weakness.<br>- Assisting with follow-up, weight goals, and timeline creation.<br>- Arranging regular follow-up visits to jointly review the progress, monitor records, manage barriers, adjust the goals, and plan as necessary.<br>- Arranging referrals for specialized nutrition, behavioural counselling, or to discuss obesity medications.
Palmeira (2019) [10]	"The present study aimed to evaluate the effect of remote nursing monitoring on the improvement of anthropometric measurements of overweight women."	**Education**<br>-Self-management.<br>-Causes and risks of obesity.<br>-Healthy eating habits.<br>**Risks to weight loss products and dieting**<br>-Physical activities.<br>-Myth busters (clarifying common misbeliefs about obesity and obesity management).<br>**Monitoring (remote)**<br>-Making regular, weekly phone calls as an adjunct to conventional treatment.

			-Providing ongoing emotional support and motivation.
Parker	2018 [11]	**Protocol** "The aim of this study is to evaluate the implementation and effectiveness of a preventive intervention in primary care structured around the 5As framework supported by a patient-facing mobile app, consultations with the PN and/or referral to a telephone coaching service."	**Proposed nursing interventions** -Using the 5As framework (assess, advise, agree, assist, arrange) during regular health visits -Promoting patient use of a purpose-built app. -Making referrals for telephone coaching.
	2022 [12]	**Trial** "The HeLP-GP trial aimed to evaluate a multifaceted intervention provided to overweight and obese patients attending primary care."	**Interventions** -Using the 5As framework and tool to perform health checks every 6 weeks (GP at 12 weeks) and to set collaborative goals. -Using an app to text appointment reminders and nutritional and activity messages each week for 6 weeks. -Making referrals for telephone counseling.
	2024 [13]	**Mixed-method** "This paper describes the experience of implementing a nurse-led obesity intervention in Australian general practice through the lens of organisational readiness."	**Professional development and ongoing support** -Liaising with primary healthcare networks to discuss and enhance intervention implementation.
Shaji et al. (2023) [14]		The aim of this study is "to determine the effectiveness of NLLMI [nurse-led lifestyle modification interventions]	**Education** -Lifestyle modifications (integrated within routine primary healthcare).

	on obesity among young women in India with an intention that nurses apply lifestyle interventions in routine care for young women with obesity".		
Virtanen (2021) [15]	"The aim of the present study was to assess the effectiveness of lifestyle counselling on weight management in a feasible setting in primary health care." The impact of weight change on quality of life was also investigated.	**Interventions**<br>-Assess baseline values (height, weight, BMI, BP, and lab tests), psychosocial risk factors, and answers from self-administered questionnaires (e.g., diet, activity, quality of life, medication, and medical history).<br>-Based on the findings, advise on lifestyle and diet goals.	**Education**<br>-Lifestyle, healthier lifestyle habits for improved quality of life.

Table S3. Preferred Reporting Items for Systematic reviews and Meta-Analyses extension for Scoping Reviews (PRISMA-ScR) checklist.

SECTION	ITEM	PRISMA-ScR CHECKLIST ITEM	REPORTED ON PAGE #
**TITLE**			
Title	1	Identify the report as a scoping review.	Page 1
**ABSTRACT**			
Structured summary	2	Provide a structured summary that includes the following (as applicable): background, objectives, eligibility criteria, sources of evidence, charting methods, results, and conclusions that relate to the review questions and objectives.	Page 1.
**INTRODUCTION**			
Rationale	3	Describe the rationale for this review in the context of what is already known. Explain why the review questions/objectives lend themselves to a scoping review approach.	Section 1. Paragraphs 3-4. Page 2.

SECTION	ITEM	PRISMA-ScR CHECKLIST ITEM	REPORTED ON PAGE #
Objectives	4	Provide an explicit statement of the questions and objectives being addressed with reference to their key elements (e.g., population or participants, concepts, and context) or other relevant key elements used to conceptualize the review questions and/or objectives.	Section 1. Page 2, bottom.
**METHODS**			
Protocol and registration	5	Indicate whether a review protocol exists; state if and where it can be accessed (e.g., a Web address), and if available, provide registration information, including the registration number.	Section 2.1. Page 3.
Eligibility criteria	6	Specify characteristics of the sources of evidence used as eligibility criteria (e.g., years considered, language, and publication status), and provide a rationale.	Section 2.2. Page 3.
Information sources*	7	Describe all information sources in the search (e.g., databases with dates of coverage and contact with authors to identify additional sources), as well as the date the most recent search was executed.	Section 2.3. Pages 3-4.
Search	8	Present the full electronic search strategy for at least one database, including any limits used, such that it could be repeated.	Section 2.4. Page 4.
Selection of sources of evidence†	9	State the process for selecting sources of evidence (i.e., screening and eligibility) included in this scoping review.	Section 2.5. Page 4.
Data charting process‡	10	Describe the methods of charting data from the included sources of evidence (e.g., calibrated forms or forms that have been tested by the team before their use, and whether data charting was performed independently or in duplicate) and any processes for obtaining and confirming data from investigators.	Section 2.6. Page 4.
Data items	11	List and define all variables for which data were sought and any assumptions and simplifications made.	Section 2.6. Page 4.
Critical appraisal of individual sources of evidence§	12	If performed, provide a rationale for conducting a critical appraisal of included sources of evidence; describe the methods used and how this information was used in any data synthesis (if appropriate).	Not performed.
Synthesis of results	13	Describe the methods of handling and summarizing the data that were charted.	Section 2.7. Page 4-5.
**RESULTS**			

SECTION	ITEM	PRISMA-ScR CHECKLIST ITEM	REPORTED ON PAGE #
Selection of sources of evidence	14	Give numbers of sources of evidence screened, assessed for eligibility, and included in the review, with reasons for exclusions at each stage, ideally using a flow diagram.	Section 3. Page 5. Plus, Figure 1.
Characteristics of sources of evidence	15	For each source of evidence, present characteristics for which data were charted and provide the citations.	Section 3.1. Page 5. Table 3. Paged 6-7.
Critical appraisal within sources of evidence	16	If performed, present data on critical appraisal of included sources of evidence (see item 12).	Not performed.
Results of individual sources of evidence	17	For each included source of evidence, present the relevant data that were charted that relate to the review questions and objectives.	Appendix B.
Synthesis of results	18	Summarize and/or present the charting results as they relate to the review questions and objectives.	Section 3.2. Pages 8-10.
**DISCUSSION**			
Summary of evidence	19	Summarize the main results (including an overview of concepts, themes, and types of evidence available), link to the review questions and objectives, and consider the relevance to key groups.	Section 4. Pages 11-13.
Limitations	20	Discuss the limitations of the scoping review process.	Section 4.1. Page 13.
Conclusions	21	Provide a general interpretation of the results with respect to the review questions and objectives, as well as potential implications and/or next steps.	Section 5. Page 13.
**FUNDING**			
Funding	22	Describe sources of funding for the included sources of evidence, as well as sources of funding for the scoping review. Describe the role of the funders of the scoping review.	Page 13.

JBI = Joanna Briggs Institute; PRISMA-ScR = Preferred Reporting Items for Systematic reviews and Meta-Analyses extension for Scoping Reviews.

* Where *sources of evidence* (see second footnote) are compiled from, such as bibliographic databases, social media platforms, and Web sites.

† A more inclusive/heterogeneous term used to account for the different types of evidence or data sources (e.g., quantitative and/or qualitative research, expert opinion, and policy documents) that may be eligible in a scoping review as opposed to only studies. This is not to be confused with *information sources* (see first footnote).
‡ The frameworks by Arksey and O'Malley (6) and Levac and colleagues (7) and the JBI guidance (4, 5) refer to the process of data extraction in a scoping review as data charting.
§ The process of systematically examining research evidence to assess its validity, results, and relevance before using it to inform a decision. This term is used for items 12 and 19 instead of "risk of bias" (which is more applicable to systematic reviews of interventions) to include and acknowledge the various sources of evidence that may be used in a scoping review (e.g., quantitative and/or qualitative research, expert opinion, and policy document).

From: Tricco AC, Lillie E, Zarin W, O'Brien KK, Colquhoun H, Levac D, et al. PRISMA Extension for Scoping Reviews (PRISMAScR): Checklist and Explanation. Ann Intern Med. 2018;169:467–473. doi: 10.7326/M18-0850.

Table S4. Extracted data items related to nurses' role in obesity management.

	Patient-centred Care						Patient Assessment													Therapeutic Interventions														Care Management								Patient Education										Professional Development					
											Lifestyle Factors					Physical Health				Interventions				Approach				Goals of Care/ Outcome Measures														Disease			Lifestyle Factors				Available Treatments			Reflexivity		Ongoing Learning		Tools	
First Author (year) Reference	[illegible]	[illegible]	[illegible]	[illegible]	[illegible]	Family / Social Support	Anthropometric	Medical Background	Readiness for Change	Mental Health	SDOH	Nutrition	Physical Activity	Smoking or Alcohol	Behaviours/ Lifestyle Habits	Comorbidities	Health Risks	Pain	Cardiometabolic Factors	Lifestyle Behaviour Δ	Supportive Care (empower/	Anti- Obesity Medication	Metabolic Surgery- Refer	[illegible]	M.I	5As	Risk Reduction (of complica-	Patient Goals	Anthropometric	QOL	Emotional Wellness	Prevent or ↓Comorbidities	Lifestyle/ behaviour Δ	Health Plan / Goal	Monitoring	Remote Monitoring	Coordinate Care	Program Development	Run Group	Schedule Or Follow-Up	Refer	Etiology/ Pathophysiology	Obesity Risks	Prevent or Treat Comorbidi-	Diet / nutrition/ drive /cues	Physical Activity	Smoking	Alcohol	Lifestyle Behaviour Δ	Devices /technology	Resources	Identify own Biases	Gaps in Knowledge	Available Guidelines	Obesity Education	5 As	M.I.
Barrea (2021) [1]	X	X			X	X	X	X	X	X	X	X	X	X	X	X			X	X	X	X		X	X		X	X	X			X	X	X	X	X				X		X	X	X	X	X	X	X	X						X		

Braga (2020) [2]			X		X		X					X				X	X			X	X		X	X					X				X		X		X	X		X	X				X	X							X	X	X		
Brewah (2018) [3]		X		X			X			X										X	X																								X			X	X		X			X	X		
Campbell-Scherer (2019) [4]*	X	X	X	X			X	O	X	O	O	O				O	X			X				X		X		X		X			X	X						X					X								X		X	X	
Fernandez-Ruiz (2018) [5]		X			X		X				X								X	X	X			X					X			X		X	X		X	X	X	X				X	X				X								
Fernández-Ruiz (2018) [6]						X	X			X			X	X		X		X	X		X			X					X	X	X	X		X	X		X	X																			
Go-vindasamy (2023) [7]		X	X				X					X								X	X							X	X				X	X	X	X				X					X												
Hinks (2022) [8]							X		X													X	X		X				X					X	X						X		X		X	X				X	X		X		X		X
Kelley (2018) [9]	X	X		X		X	X					X			X					X	X	X				X	X		X				X	X	X			X	X	X	X		X		X	X			?			X				X	
Palmeira (2019) [10]					X		X				X	X	X	X	X	X				X	X						X		X			X	X		X	X			X	X		X	X	X	X	X				X				X			
Parker (2018) [11]		X					X					X	X				X		X	X	X					X	X			X			X	X	X					X	X		X		X	X	X		X	X				X	X		
Parker (2022) [12]		X	X				X					X	X		X					X						X			X	X		X	X	X	X					X	X				X	X									X		
Parker (2024) [13]		X	X				X										X			X					X	X		X					X	X	X		X			X	X		X		X	X								X	X		X
Shaji (2023) [14]							X				X	X	X	X	X					X					X		X		X	X		X	X		X	X				X					X	X			X								
Virtanen (2021) [15]		X					X	X		X	X	X	X	X	X	X			X	X									X	X															X												

Table S5. Specific patient assessments and education discussed by primary care nurses.

	PATIENT ASSESSMENT OF:																										PATIENT EDUCATION ON:											
	Back-ground			Mental					Lifestyle										Anthropo-metric			Physical Health					Disease				Lifestyle				Treatment Op-tions			
First Author (year) Reference	Medical History	Medication History	Root Causes	Depression	Anxiety	Body Image	Stress	Psychotropic Medication	Smoking	Behaviours/ Habits	Physical Activity	Social Isolation	Sleep	Nutrition	Alcohol	SDOH	QOL	Ready For Change?	Weight Only	BMI (Weight & Height)	Waist Circumference	Comorbidities	Blood Pressure	Pain	Health Risks	Cardiometabolic Factors	Etiology/ Pathophysiology	Prevention &/or Treatment of [illegible]	Obesity Health Risks	Root Causes	Physical Activity	Smoking	Nutrition	Alcohol	" Lifestyle Behaviors"	Resources	Technology	Say " Lose Weight"
Barrea (2021) [1]	X	X	X	X	X	X	X			X	X		X	X	X	X		X		X	X	X	X			X	X	X	X	X	X	X	X	X	X			
Braga (2020) [2]														X						X	X	X	X		X						X		X					
Brewah (2018) [3]							X													X													X	X	X	X		

Campbell-Scherer (2019) [4]*		O		O	O	O	X		O						O		O		O		X		O			X								O					
Fernandez-Ruiz (2018) [5]																	X				X	X		X			X		X					X		X			
Fernández-Ruiz (2018) [6]						X				X		X				X		X			X		X		X		X												
Govindasamy (2023) [7]															X					X														X					
Hinks (2022) [8]																			X		X									X		X		X			X	X	
Kelley (2018) [9]											X				X						X									X		X		X		?			X
Palmeira (2019) [10]										X	X	X			X	X	X				X	X	X					X	X	X		X		X				X	
Parker	(2018) [11]											X			X						X	X		X		X	X			X		X	X	X		X		X	X
	(2022) [12]										X	X			X			X			X	X		X								X		X					X
	(2024) [13]																				X	X				X				X		X		X				X	
Shaji (2023) [14]										X	X	X		X	X		X				X											X		X		X			
Virtanen (2021) [15]			X		X	X		X		X	X	X	X	X	X	X	X	X			X		X	X			X							X					X

References

1. Barrea, L.; Framondi, L.; Di Matteo, R.; Verde, L.; Vetrani, C.; Graziadio, C.; Pugliese, G.; Laudisio, D.; Vitale, G.; Iannicelli, A.; et al. The role of the nurse in the Obesity Clinic: a practical guideline. *Panminerva Medica* **2021**, *63*, doi:10.23736/s0031-0808.21.04540-7.
2. Braga, V.A.S.; Jesus, M.C.P.; Conz, C.A.; Silva, M.H.D.; Tavares, R.E.; Merighi, M.A.B. Actions of nurses toward obesity in primary health care units. *Rev Bras Enferm* **2020**, *73*, e20180404, doi:10.1590/0034-7167-2018-0404.
3. Brewah, H.; Todhunter, J.; Bades, A.; Colyer, S. Can community nurses take on obesity? *Journal of Community Nursing* **2018**, *32*, 8-11.
4. Campbell-Scherer, D.L.; Asselin, J.; Osunlana, A.M.; Ogunleye, A.A.; Fielding, S.; Anderson, R.; Cave, A.; Johnson, J.A.; Sharma, A.M. Changing provider behaviour to increase nurse visits for obesity in family practice: the 5As Team randomized controlled trial. *CMAJ Open* **2019**, *7*, E371-E378, doi:10.9778/cmajo.20180165.
5. Fernández-Ruiz, V.E.; Armero-Barranco, D.; Paniagua-Urbano, J.A.; Sole-Agusti, M.; Ruiz-Sánchez, A.; Gómez-Marín, J. Short-medium-long-term efficacy of interdisciplinary intervention against overweight and obesity: Randomized controlled clinical trial. *Int J Nurs Pract* **2018**, *24*, e12690, doi:10.1111/ijn.12690.
6. Fernández-Ruiz, V.E.; Paniagua-Urbano, J.A.; Solé-Agustí, M.; Ruiz-Sánchez, A.; Gómez-Marín, J.; Armero-Barranco, D. Impact of the I(2)AO(2) interdisciplinary program led by nursing on psychological comorbidity and quality of life: Randomized controlled clinical trial. *Arch Psychiatr Nurs* **2018**, *32*, 268-277, doi:10.1016/j.apnu.2017.11.014.
7. Govindasamy, S.; Beek, K.; Yates, K.; Jayasuriya, R.; Reynolds, R.; de Wit, J.B.F.; Harris, M. Experiences of overweight and obese patients with diabetes and practice nurses during implementation of a brief weight management intervention in general practice settings serving Culturally and Linguistically Diverse disadvantaged populations. *Aust J Prim Health* **2023**, *29*, 358-364, doi:10.1071/PY22013.
8. Hinks, S.R. Exploring community nurses' views on the implementation of a local Weight Management Pathway. *British journal of community nursing 27*, 612-618, doi:10.12968/bjcn.2022.27.12.612.
9. Kelley, S. The Role of the Faith Community Nurse in Weight Management. *J Christ Nurs* **2018**, *35*, 53-59, doi:10.1097/cnj.0000000000000449.
10. Palmeira, C.S.; Mussi, F.C.; Santos, C.A.S.d.T.; Lima, M.L.; Ladeia, A.M.T.; Silva, L.C.d.J. Effect of remote nursing monitoring on overweight in women: clinical trial. *Revista Latino-Americana de Enfermagem* **2019**, e3129, doi: 10.1590/1518-8345.2651.3129.
11. Parker, S.M.; Stocks, N.; Nutbeam, D.; Thomas, L.; Denney-Wilson, E.; Zwar, N.; Karnon, J.; Lloyd, J.; Noakes, M.; Liaw, S.-T.; et al. Preventing chronic disease in patients with low health literacy using eHealth and teamwork in primary healthcare: protocol for a cluster randomised controlled trial. *BMJ Open* **2018**, *8*, e023239, doi:10.1136/bmjopen-2018-023239.
12. Parker, S.M.; Barr, M.; Stocks, N.; Denney-Wilson, E.; Zwar, N.; Karnon, J.; Kabir, A.; Nutbeam, D.; Roseleur, J.; Liaw, S.-T.; et al. Preventing chronic disease in overweight and obese patients with low health literacy using eHealth and teamwork in primary healthcare (HeLP-GP): a cluster randomised controlled trial. *BMJ Open* **2022**, *12*, e060393, doi:10.1136/bmjopen-2021-060393.
13. Parker, S.; Tran, A.; Saito, S.; McNamara, C.; Denney-Wilson, E.; Nutbeam, D.; Harris, M.F. Exploring organisational readiness to implement a preventive intervention in Australian general practice for overweight and obese patients: key learnings from the HeLP-GP trial. *Australian Journal of Primary Health* **2024**, *30*, doi:10.1071/py23085.
14. Shaji, P.; Singh, M.; Sahu, B.; Arulappan, J. Effectiveness of Nurse-led Lifestyle Modification Intervention on Obesity Among Young Women in India. *SAGE Open Nursing* **2023**, *9*, doi:10.1177/23779608231186705.
15. Virtanen, J.; Penttinen, M.; Kautiainen, H.; Korhonen, P. The impact of lifestyle counselling on weight management and quality of life among working-age females. *Scand J Prim Health Care* **2021**, *39*, 382-388, doi:10.1080/02813432.2021.1958510.
